# Combining two injectable progesterone formulas for estrous synchronization in ewes

**DOI:** 10.1590/1984-3143-AR2024-0073

**Published:** 2024-10-07

**Authors:** Milena Luzorio Simões, Felipe Zandonadi Brandão, Juliana Dantas Rodrigues Santos, Pedro Henrique Nicolau Pinto, Ana Paula Pereira Schmidt, Camila Correa Roza Laeber, Nathália Dutra Knust, Mariana Garcia Kako Rodriguez, Danilo Fila, María Isabel Vázquez, Rodolfo Ungerfeld

**Affiliations:** 1 Faculdade de Veterinária, Universidade Federal Fluminense, Niterói, RJ, Brasil; 2 Departamento de Biociencias Veterinarias, Facultad de Veterinaria, Universidad de la República, Montevideo, Uruguay; 3 U.A. Reproducción Animal, Departamento de Producción Animal y Salud de los Sistemas Productivos, Facultad de Veterinaria, Universidad de la República, San José, Uruguay

**Keywords:** follicle dynamics, reproductive seasonality, reproductive efficiency, sheep

## Abstract

The study aimed to evaluate the effectiveness of combining two injectable progesterone (iP4) formulas for estrous synchronization in ewes and to compare it with traditional intravaginal progesterone devices. Additionally, the study assessed whether the inclusion of GnRH enhances the reproductive outcomes of the iP4 treatment. Two experiments were conducted. In the first experiment, 20 Santa Inês ewes were divided into two groups: one group received intravaginal progesterone devices, and the other received combined long-acting and short-acting iP4. In the second experiment, 30 Corriedale ewes were divided into two groups: one received the combined iP4 with GnRH, and the other without GnRH. Estrous, ovulation, follicular populations, and progesterone concentrations were monitored. The combined iP4 treatment induced an artificial luteal phase and produced reproductive responses similar to those obtained with intravaginal devices. In the first experiment, the iP4 treatment tended to result in more synchronized ovulation compared to the control (P=0.095). In the second experiment, adding GnRH enhanced the quality of the corpus luteum, as indicated by increased diameter and vascularization on Day 23 (P=0.047 and P=0.02, respectively). The combined administration of long-acting and short-acting iP4 effectively synchronized estrous in ewes and showed similar efficacy to traditional intravaginal devices. The inclusion of GnRH improved luteal quality, suggesting potential benefits for reproductive management in ewes. Further studies are needed to evaluate the fertility outcomes of these protocols under field conditions.

## Introduction

The basis for hormonal treatments for handling the estrous cycles in small ruminants has been developed in the last 70 years (Gonzalez-Bulnes et al., 2020). The most widely used treatments are based on the insertion of intravaginal devices impregnated with progesterone or analogues, which are easy to use and provide effective results. In general, intravaginal devices are made of silicone (e.g.: CIDR; Controlled Internal Drug Release) or sponges of polyurethane impregnated with progestogens (medroxyprogesterone acetate, MAP; flurogestone acetate, FGA) [reviewed by Cosentino et al., 2023]. However, in vaginal and cervical artificial insemination procedures, fertility diminishes after the use of these devices (Manes et al., 2014), which may be explained by changes in the vaginal microbiota, vaginitis (Manes et al., 2018), and an inflammatory process in the mucosa (Manes et al., 2015). All these factors modify the local environment, affecting sperm quality (Manes et al., 2016). In addition, the devices can promote changes in the female's odor, leading to a decrease in the male's sexual attraction (Gatti and Ungerfeld. 2012). Another important issue that remains unresolved is the high amount of hormonal residue remaining in the device, which is usually discarded and may act as an environmental disruptor (Liu et al., 2015; Sauer et al., 2018). An alternative to avoid these problems is the use of injectable progesterone (iP4), as all the hormone administrated enters through the same metabolic pathways as the secreted hormone. While this approach was discarded 50 years ago due to its short half-life (Robinson, 1956) long-lasting formulas have recently been developed and are available on the market, opening the possibility of overcoming the requirement of repeated administrations (D’Ávila et al., 2022; Salarpoor et al., 2023).

The use of iP4 has been tested in bovines (Goletiani et al., 2007), but there is still scarce information on its potential use in sheep. The mechanism of action of this hormonal formulation needs to be better elucidated to standardize factors such as the route of administration, dose used, and application frequency. A main issue is that the progesterone drop should be sufficiently synchronized to fall below luteal levels in all the ewes in a short period of time. Recently, blood concentration patterns of two injectable progesterone formulations (short and long-acting) have been described, maintaining luteal concentrations for more than 24 hours (short-acting) and 5 days (long-acting) regardless of the doses used (Santos et al., 2022, 2023). However, although the long-acting formula may be of more practical use, as expected, the reduction to basal concentrations is more dispersed over time than is the case with the short-acting formulation.

Based on this information, we hypothesize that the combined administration of the long-acting formula, followed 5 days later by the administration of the short-acting formula, allows for the maintenance of an artificial luteal phase for at least 5-6 days, followed by a synchronized reduction in progesterone concentrations. What would be of great usage for developing synchronization protocols without intravaginal implants. Thus, the objective of the first study was to compare the progesterone profile, follicular populations, and reproductive responses between the combined treatment and the use of a traditional progesterone intravaginal device in ewes. Next, as the response to the iP4-based treatment achieved similar results to those provided with the intravaginal device, the aim of the second study was to determine if the inclusion of GnRH to synchronize the beginning of the ovulatory follicular wave enhances the reproductive results of the iP4 treatment.

## Methods

The experiments were approved by the Ethics Commission on the Use of Animals (CEUA) of the Universidade Federal Fluminense, Brazil (protocol 7723011221) and Facultad de Veterinaria, Universidad de la República, Uruguay (protocol 1676/23).

### Experimental location, animals and facilities

Two experiments were evaluated; one was performed at the Unidade de Pesquisa Experimental em Caprinos e Ovinos of the Universidade Federal Fluminense, located in Cachoeiras de Macacu (22°S, 42°W), Rio de Janeiro, Brazil, and the second was carried out at Estación Experimental Nº 1, Facultad de Veterinaria, Universidad de la República, located in Migues, Canelones, Uruguay (34° 22' S, 55° 36' W). The animals used in the studies were previously evaluated and only healthy animals with no reproductive abnormalities were included in the experiments. Regardless of their reproductive status (cycling or not), ewes were included in the study, but this number was balanced between treatments.

### Experiment 1

The experiment included 20 multiparous Santa Inês ewes weighing 48.1 ± 3.1 (mean ± SEM) and with a body condition score of 2.8 ± 0.1 (1-5 scale) (Suiter, 1994). The experiment was performed during February (mid-summer, breeding season). The animals were maintained in an intensive system with capiaçu grass (*Pennisetum purpureum* Schumach) and concentrate (18% crude protein and 75% total digestible nutrients), with free access to water and mineral salt.

**Figure 1:**
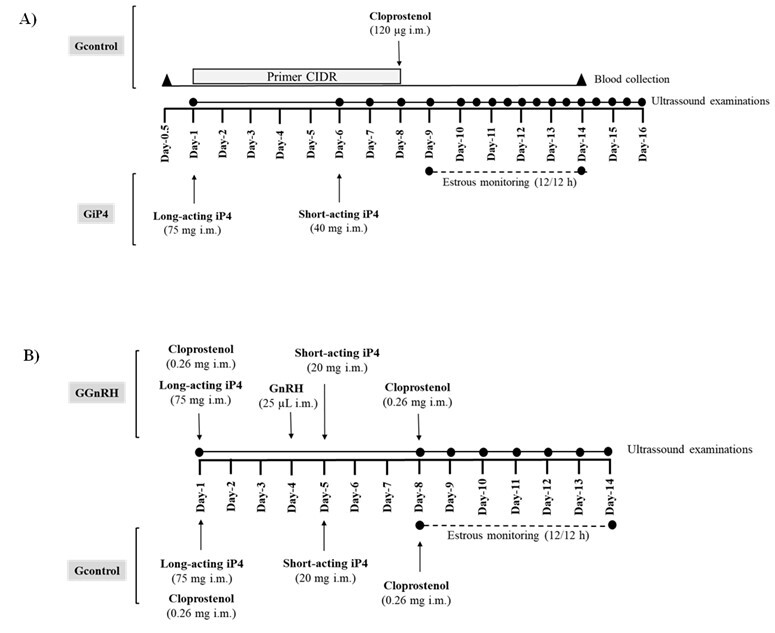
Schematic representation of the first (A) and second (B) experimental procedures. iP4: injectable progesterone. i.m.: intramuscular

Ewes were allocated to two experimental treatments (n=10/each). On Day 1, an intravaginal device containing natural progesterone (0.36 g; Primer PR – União Química, São Paulo, Brazil) was inserted into 10 ewes (Gcontrol1), remaining in situ for 7 days. The remaining 10 ewes (GiP4) received a single dose of long-acting iP4 (75 mg; i.m.; Biorelease - Technologies LLC of Lexington, USA) on Day 1, followed by a single dose of short-acting injectable progesterone (40 mg; i.m.; Progecio – 7% progesterone - União Química, São Paulo, Brazil) on Day 6, both administered i.m. On Day 8, cloprostenol (0.26 mg. i.m.; Estron, Agner União, São Paulo, Brazil) was administered to all ewes. Beginning on Day 9, sexual receptiveness (see Assessment of sexual behavior section) was monitored every 12 h until Day 14. Ovarian ultrasound evaluations were performed daily (see Ultrasound evaluations section) from Day 6 to Day 16. Blood was also collected daily from Day 0.5 to Day 14 to measure progesterone plasma concentrations (see Blood collection and plasma P4 concentration section) (Figure 1A).

### Experiment 2

The experiment was performed in March (late summer, breeding season) with 30 multiparous Corriedale ewes. During the study, the animals remained in an extensive grazing system on native pastures, with *ad libitum* access to water.

The ewes were allocated to two experimental groups of 15 ewes each, with two repetitions (n=15 in each, 5 from each treatment). On Day 1, with ewes on a random day of their estrous cycle, 75 mg of the same long-acting injectable progesterone formula plus 0.26 mg of a PGF2alpha analogue (cloprostenol, Estron, Agener União, São Paulo, Brazil) were i.m. administered. On Day 4, 15 ewes received a GnRH dose (25 µl, i.m.; Tec-Relin, Agener União, São Paulo, Brazil - GGnRH), and the other 15 ewes received no other treatment (Gcontrol2). On Day 5, all ewes received a single dose of 20 mg of the short-acting iP4, i.m. administered. Subsequently, on Day 8, all ewes received a second dose of cloprostenol (0.26 mg, i.m.; Dalmaprost-D, Fatro Fedagro S.R.L., Montevideo, Uruguay). Sexual receptiveness was monitored every 12 h, and ovarian follicular populations were recorded by ultrasound assessments from Day 8 to Day 15 (Figure 1B).

### Assessment of sexual behavior

In both experiments, sexual receptiveness was determined twice daily with adult experienced rams. The rams were fitted with a protective apron affixed to their abdominal region to prevent copulation at the time of teasing. Estrous detection was performed in groups of five ewes, and the rams were replaced if they showed fatigue. An ewe was considered to be receptive if she remained immobile while the ram was mounting her.

### Ultrasound evaluations

B-mode ultrasound evaluations were performed to record the ovarian follicular populations every 12 h in all animals, until the time of ovulation was confirmed or until Day 8 (Experiment 1) or Day 10 (Experiment 2). A portable ultrasound device (SonoScape, Model S 6, Shenzhen, China) was used, with a 7.5 MHz transrectal linear probe attached to a PVC guide; the device was slightly curved and adapted for small ruminants. The ewes were restrained in trunks, remaining on station during the procedure. With the aid of a 60 mL syringe, a small amount of gel (Carbogel UTL, Carbogel Indústria e Comércio LTDA, São Paulo, Brazil) was inserted into the rectum of the ewes. After introducing the transducer and locating the bladder, the evaluation of the ovaries began, counting the number of small (≤ 2-3 mm), medium (3-5 mm), and large (≥ 5 mm) follicles. A follicle was considered to have ovulated after confirmation of its disappearance for two consecutive examinations, confirmed later by the presence of a corpus luteum (CL). The presence, number and diameter of the CLs were determined on Days 17 and 23. The functionality of the CLs was evaluated with color Doppler ultrasound on Day 23 using pixel evaluations and vascularization area of CLs. The images were video recorded, and the images of the CLs were analyzed after identifying the image with the greatest perfusion. These images were analyzed with Image J Ops, measuring the vascularization area using the CTRLM function, the selection of the area with the colored pixels (to be evaluated) was performed manually. As the perfused areas were separated in some images, the sum of all those areas was considered as the area vascularized of the total CL area.

### Blood collection and plasma P4 concentration

Blood samples were collected by jugular venipuncture. The first two blood samples were collected at 12-hour intervals and after collected every 24 hours, from Day 1 to Day 15, in Experiment 1. Blood samples were collected in tubes with EDTA and centrifuged at 1,500 g for 15 min, and the plasma was then separated and frozen at -20 ^◦^C. Progesterone concentrations were measured by solid-phase radioimmunoassay using commercial kits (MP Diagnostics Division; Orangeburg, NY, USA) in a single assay to avoid inter-assay variability. The detection limit was 0.15 ng/mL, and the intra-assay coefficient of variation was 8%. All data were within the minimum and maximum values of the curve.

### Definition of variables and statistical analysis

The interval from the administration of cloprostenol to the onset of estrus and to the time of ovulation, the estrous length, the proportion of ewes that came into estrus and that ovulated, the number of ovulations per ewe, and the diameter of the largest ovulatory and non-ovulatory follicles were considered for the analysis. In Experiment 1, the percentage of ewes that ovulated and the number of ovulations were also included in the analysis.

The proportion of ewes that came into estrus and of ewes that ovulated were compared using the Fisher exact probability test. The other data were analyzed using mixed models with SAS statistical software (SAS on Demand for Academics). The models included treatment as the main effect and repetitions as a random factor. The dispersion of the time of ovulations was compared using Bartlett’s test. The follicular populations, the diameter of the largest and second largest follicles, and the progesterone concentrations were analyzed using the mixed procedure including the treatments, time, and their interactions as the main effects, with time as a repeated measure, and repetitions as a random effect. As ovulation occurs on different moments after the treatment, data were normalized to the moment of the application of the treatments, but not to the postovulatory moment. Therefore, only the follicular data from those ewes that ovulated, and only until the time of ovulation were included in the analysis. Data are presented as LS mean ± SEM. For all tests, differences were considered significant when P ≤ 0.05 and as a tendency when 0.05 < P ≤ 0.1.

## Results

### Experiment 1

The interval from the cloprostenol administration to the onset of estrus tended to be longer in the GiP4 ewes than in the Gcontrol1 ewes (P=0.06), with no differences in the other evaluated reproductive responses. The treatment with iP4 tended to induce an ovulation response that was more concentrated (P=0.095) (Table 1).

**Table 1:** Reproductive responses of Santa Inês ewes subjected to a hormonal protocol based on the combination of two injectable formulas of progesterone (GiP4) or an intravaginal device impregnated with progesterone for 7 days (Gcontrol1). Both groups received 120 µg of cloprostenol on Day 8 of the protocol.

**Reproductive responses**	**GiP4**	**Gcontrol1**	**P**
Estrous manifestation rate (%)	80.0% (8/10)	90.0% (9/10)	ns
Interval from CLO to estrus onset (h)	68.5 ± 5.9	52.2 ± 5.4	0.06
Estrous length (h)	36.7 ± 5.1	42.6 ± 5,31	ns
Ewes that ovulated (%)	80.0% (8/10)	90.0% (9/10)	ns
Interval from CLO to ovulation (h)^1^	106.6 ± 4.6	99.6 ± 8.5	ns
Ovulation (number)	1.0 ± 0.0	1.0 ± 0.0	ns
Diameter of the largest follicle (mm)	5.0 ± 0.3	5.5 ± 0.2	ns

Values are presented as LS mean ± standard error of the mean. ns: not significant; CLO: cloprostenol. ^1^ Dispersion of the data: P=0.095.

**Figure 2:**
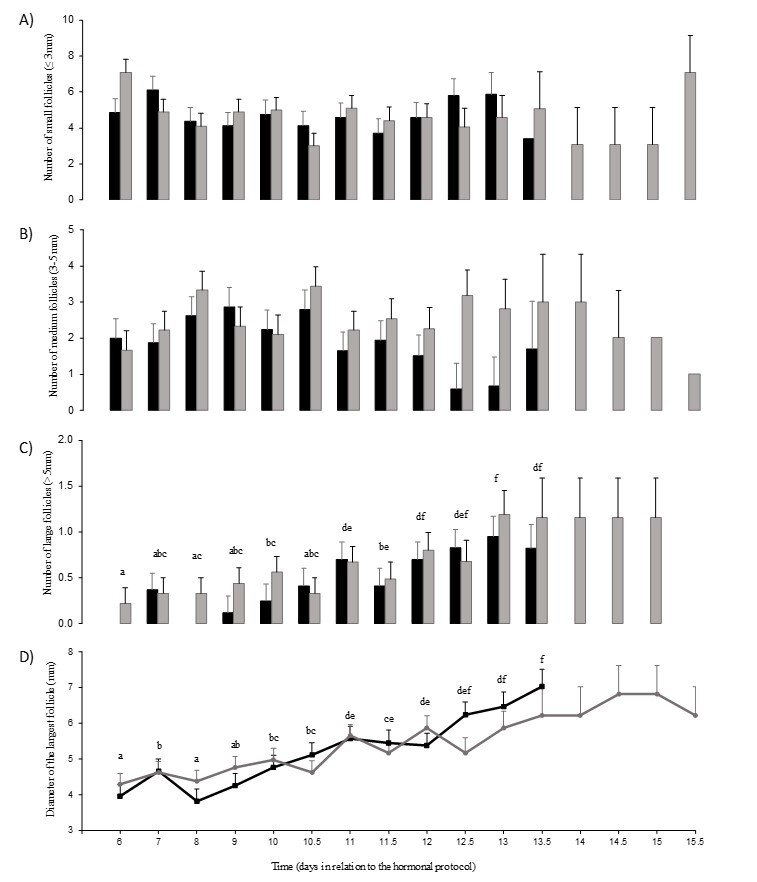
LS mean (± s.e.m.) population of (A) small (> 5 mm), (B) medium (3–5 mm), and (C) large (< 3 mm) follicles, and diameter of the largest (D) follicle of Santa Inês ewes subjected to a hormonal estrous synchronization protocol based on a combination of progesterone (long and short-action, see text for more details, GiP4, black bars) or a traditional protocol with intravaginal implant impregnated with progesterone (gray bars, Gcontrol1). Differences observed in time, independent of treatment, are indicated with different letters (P≤0.05). The interval from the administration of the dose of cloprostenol to the time of ovulation (h) was 68.5 ± 5.9 and 52.2 ± 5.4, in GiP4 and Gcontrol1, respectively.

The follicular populations of small and medium-size follicles did not vary with treatments, time, or their interaction, although that of medium follicles tended to vary with time (P=0.08) (Figures 2A and 2B). The follicular population of large follicles, and the size of the greatest follicle were not affected by treatments or the interaction between treatment and time but varied with time (P<0.0001 for both variables; Figures 2C and 2B). The number of large follicles was greater on Days 10, 11, 12 and thereafter the values were greater than the initial ones (P<0.05). From Day 12.5 the number was greater than that observed before Day 10.5 (P<0.05) (Figure 2C). The diameter of the largest follicle increased on Day 10, again on Day 11, and again on Day 13 (P<0.05, Figure 2D).

**Figure 3:**
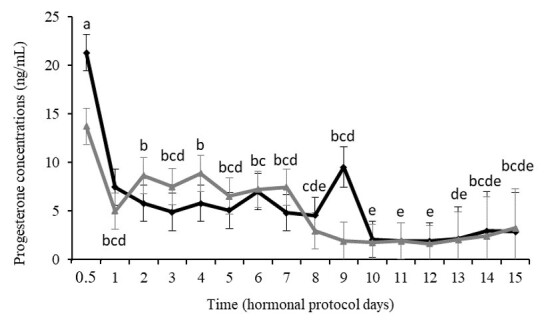
Plasmatic progesterone concentrations in Santa Inês ewes subjected to the hormonal estrus synchronization protocol based on a combination of progesterone (long and short-action, see text for more details, GiP4, black line) or the traditional protocol with intravaginal implant impregnated with progesterone (gray line, Gcontrol1). Differences observed in time, independent of treatment, are indicated with different letters (P≤0.05).

Plasma progesterone concentrations varied with time, and there was a tendency for an interaction between treatment and evaluation time (P<0.0001 and P=0.08, respectively) (Figure 3). There was a peak in plasma progesterone concentrations 12 h after the administration of iP4 or after insertion of the device. From this period onwards, concentrations progressively decreased throughout the evaluations, with a more marked reduction on Day 8 and between Days 10 and 15, although the levels remained at luteal concentrations until the end of the sampling period (Figure 3).

### Experiment 2

Estrus tended to be longer in the GGnRH ewes than in the Gcontrol2 ewes (P=0.06), with no other differences between the treatments for any reproductive response (Table 2).

**Table 2:** Reproductive parameters of Corriedale ewes subjected to a hormonal protocol based on the combination of two injectable formulas of progesterone associated (GGnRH) or not (Gcontrol2) with GnRH.

**Reproductive responses**	**Gcontrol2**	**GGnRH**	**P**
Estrous manifestation rate (%)	67.0% (10/15)	33.0% (5/15)	0.058
Interval from second CLO to estrus onset (h)	39.3 ± 8.0	37.2 ± 10.7	ns
Estrous length (h)	43.8 ± 5.7	45.6 ± 8.0	0.058
Ewes that ovulated (%)	100.0% (15/15)	100.0% (15/15)	ns
Interval from second CLO to ovulation (h)	55.0 ± 17.8	61.0 ± 12.4	ns
Interval from estrus onset to ovulation (h)	18.0 ± 16.5	18,0 ± 15.5	ns
Diameter of the largest ovulatory follicle (mm)	5.2 ± 0.2	5.4 ± 0.2	ns
CL diameter on Day 17 (mm)	9.9 ± 1.0	10.5 ± 1.4	ns
CL diameter on Day 23 (mm)	10.3 ± 1.3	11.3 ± 1.2	0.047
CL vascularization area on Day 23 (mm^2^)	0.4 ± 0.1	0.7 ± 0.1	0.02
CL Pixel Color Doppler Mode on Day 23	0.3 ± 0.1	0.3 ± 0.1	ns

Values are presented as LS mean ± standard error of the mean. ns: not significant; CLO: cloprostenol.

**Figure 4:**
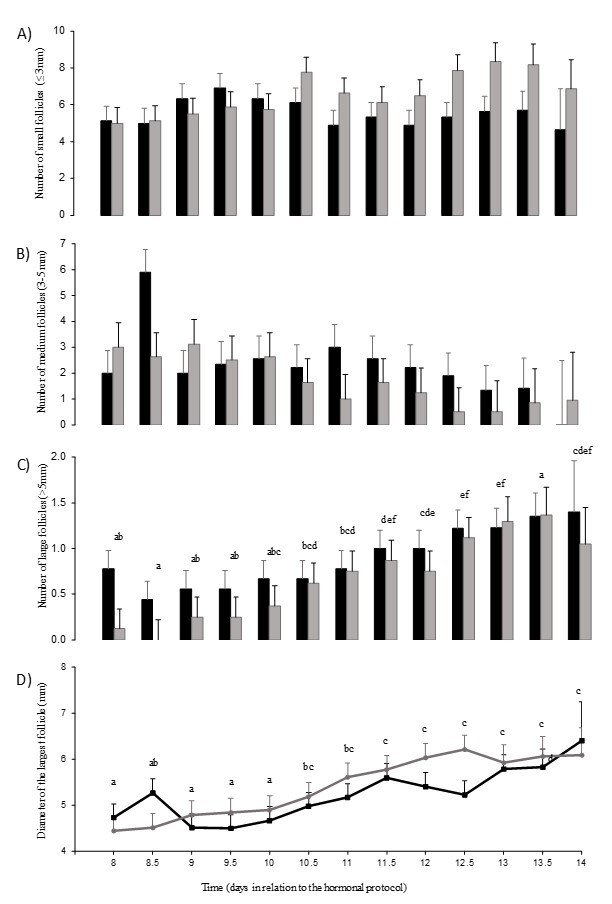
LS mean (± s.e.m.) population of (A) small (> 5mm), (B) medium (3–5 mm) and (C) large (> 5 mm) follicles, and diameter of the largest (D) follicle of Santa Inês ewes subjected to a hormonal estrus synchronization protocol based on a combination of progesterone (short and long-action) with (GGnRH, black bars) or without GnRH (Gcontrol2, gray bars), see text for more details. Means with different letters are significantly different (P > 0.05). The interval from administration of the dose of cloprostenol to the time of ovulation (h) was 55.0 ± 16.5 and 61.0 ± 12.4, for Gcontrol2 and GGnRH, respectively.

The follicular populations of small and medium-size follicles did not vary with treatments, time, or their interaction, although the number of medium follicles tended to vary with time (P=0.07) (Figures 4A and 4B). The follicular population of large follicles, and the size of the greatest follicle were not affected by treatments or the interaction between treatment and time, but varied with time (P<0.0001 for both variables; Figures 4C and 4B). The number of large follicles increased from Day 9.5 to Day 10.5, again on Day 12.5, reaching the greatest number on Day 13.5 (P<0.05) (Figure 4C). The diameter of the largest follicle increased from Day 10 to Day 10.5 (P<0.05), remaining stable from thereafter (Figure 4D).

The diameter of the CL was similar among treatments on Day 17, but greater in the GGnRH ewes than in the Gcontrol ewes on Day 21 (P = 0.047). The area of the CL vascularised was greater in the GGnRH ewes than in the Gcontrol ewes on Day 21 (P = 0.02) (Table 2).

## Discussion

The results demonstrated that the administration of a long-acting iP4 followed by a short-acting iP4 five days later, associated with the administration of cloprostenol, produced an artificial luteal phase and induced some reproductive responses similar to those observed with a commercial silicone device impregnated with progesterone. This treatment is based on the use of a long-acting iP4 to maintain the progesterone luteal levels for enough time to expect good reproductive responses (Ungerfeld and Rubianes 2019), avoiding the decrease of values due to the administration of a short-acting formula. At the same time, the progesterone concentrations induced by this iP4 receded in a more synchronized fashion (Santos et al., 2022), inducing a reproductive response similar to that obtained with the use of the silicone device. Overall, this treatment overcame the limitation of the short lifespan of traditional progesterone formulations without increasing the variation of the reproductive responses. Moreover, the time of ovulation tended to be even more synchronized than after the use of the commercial progesterone-based intravaginal device. In this context, these results are promising and pave the way for developing new protocols without the need for intravaginal devices. Avoiding the use of intravaginal devices can be advantageous for sperm quality, since the cervical and vulvar secretions produced in response to the presence of the sponge can negatively influence sperm viability (Manes et al., 2016) influencing the pregnancy rate (Manes et al., 2014). Similarly, this can potentially substitute the use of intravaginal devices, avoiding the possible contamination of the steroid content remaining in the used devices (Martin et al., 2004). Therefore, the treatment is encouraging, allowing us to study the reproductive responses deeper, although it is necessary to be cautious as the final endpoint – fertility – was not evaluated here.

The progesterone profile achieved with this treatment, combining both iP4 formulas, was similar to that obtained with the commercial device and, therefore, promotes a similar follicular development. In this sense, it is relevant that any variable of the follicular populations or its’ size differed between the treatments, therefore, reinforcing the similarity of the effects of both treatments. Although the follicular wave dynamics during the treatment were not studied in these experiments, it is well known that the follicular wave turnover and therefore, the lifespan of dominant follicles – is mainly regulated by progesterone concentrations (Driancout, 2001). This is crucial for expecting good fertility with this treatment, as the stage of follicular growth at the time of luteolysis – or progesterone withdrawn in artificial luteal phases – is related to the expected fertility (Gonzalez-Bulnes et al., 2020). Therefore, reinforcing the previous outcome, having a treatment that facilitates the mimicking of the progesterone profile produced by intravaginal devices should be the basis for deeper studies.

In the first study, time of ovulation tended to be more synchronized with the treatment based on iP4 than with the commercial intravaginal device. Again, although it requires confirmation through more field studies, the treatment appears interesting for timed artificial insemination, which requires all ovulations to occur in a short period. Moreover, it is interesting that although GnRH was applied in our second study, with similar protocol, as it has been proven to be effective for synchronizing follicular wave emergence (Souza-Fabjan et al., 2017), it was ineffective in concentrating ovulations, more than in the iP4 treatment per se, demonstrating that the progesterone concentrations achieved were enough to maximize the follicular wave turnover.

Unfortunately, in the first study, the outcoming luteal phase was not evaluated, as an adequate secretion of progesterone is crucial for achieving high pregnancy rates. In the second study, the administration of GnRH enhanced the CL quality, a result that requires a deeper understanding of the possible differences in the final follicular growth of the ovulatory follicle in ewes treated with iP4. In effect, although the follicular populations were similar, as was the size of the ovulatory follicle, there were probably functional differences not evaluated in these studies that modified the luteogenic process. This emerges as a warning light since, as has been sponges (Pérez-Clariget et al., 2021). Understanding the determinants of the difference in luteal quality more clearly may be useful for the development of complementary strategies in these treatments. Nonetheless, at least at this stage, the addition of GnRH appears to be a practical alternative to overcome this limitation.

## Conclusion

The administration of a long-acting iP4 formula followed by a single dose of a short-acting formula five days later induced an artificial luteal phase with a progesterone profile that did not differ from that induced by commercial progesterone intravaginal devices. In concordance with this, the reproductive responses and the follicular populations induced by both treatments were similar. The addition of GnRH to a similar iP4-based protocol enhanced the luteal quality outcomes. These results open up the possibility of testing these protocols to determine fertility outcomes in field conditions.
